# A Python package for fast GPU‐based proton pencil beam dose calculation

**DOI:** 10.1002/acm2.70093

**Published:** 2025-04-09

**Authors:** Mahasweta Bhattacharya, Calin Reamy, Heng Li, Junghoon Lee, William T. Hrinivich

**Affiliations:** ^1^ Department of Radiation Oncology and Molecular Radiation Sciences Johns Hopkins University Baltimore Maryland USA

**Keywords:** dose calculation, intensity modulated proton therapy, proton therapy

## Abstract

**Purpose:**

Open‐source GPU‐based Monte Carlo (MC) proton dose calculation algorithms provide high speed and unparalleled accuracy but can be complex to integrate with new applications and remain slower than GPU‐based pencil beam (PB) methods, which sacrifice some physical accuracy for sub‐second plan calculation. We developed and validated a Python package implementing a GPU‐based double Gaussian PB algorithm for intensity‐modulated proton therapy (IMPT) planning research applications requiring a simple, widely compatible, and ultra‐fast proton dose calculation solution.

**Methods:**

Beam parameters were derived from pristine Bragg peaks generated with MC for 98 energies in our clinical treatment planning system (TPS). We validated the PB approach against measurements by comparing lateral spot profiles (using single‐Gaussian sigma) and proton ranges (using R80) for pristine Bragg peaks, as well as spread‐out Bragg peaks (SOBPs) in water. Further comparisons of PB and MC from the TPS were performed in a heterogeneous digital phantom and patient plans for four cancer sites using 3D gamma passing rates and dose metrics.

**Results:**

The PB algorithm enabled dose calculation following a single Python import statement. Mean ± standard deviation (SD) errors in sigma, R80, and SOBP dose were 0.05 ± 0.01, 0.0 ± 0.1 mm, and 0.4 ± 1.1%, respectively. Mean ± SD patient plan computation time was 0.28 ± 0.07 s for PB versus 4.68 ± 2.68 s for MC. Mean ± SD gamma passing rate at 2%/2 mm criteria was 96.0 ± 5.1%, and the mean ± SD percent difference in dose metrics was 0.5 ± 3.6%. PB accuracy degraded beyond bone and lung boundaries, characterized by inaccuracies in lateral proton scatter.

**Conclusion:**

We developed a GPU‐based proton PB algorithm compiled as a Python package, providing simple beam modeling, interface, and fast dose calculation for IMPT plan optimization research and development. Like other PB algorithms, accuracy is limited in highly heterogeneous regions such as the thorax.

## 1 INTRODUCTION

Intensity‐modulated proton therapy (IMPT) is a modern radiotherapy technique consisting of narrow proton beams delivered using pencil beam scanning (PBS) systems. IMPT combines advantageous depth‐dose characteristics of protons with intensity modulation to target cancer, while reducing integral dose compared to photon radiotherapy. Many aspects of IMPT plan optimization depend on computing proton dose in tissue, including spot weight optimization, robust scenario calculation, and dose metric evaluation, and many commercial and open‐source proton dose calculation algorithms have been investigated.^1^, ^2^, ^3^, ^4^, ^5^, ^6^, ^7^, ^8^, ^9^, ^10^, ^11^, ^12^, ^13^, ^14^, ^15^, ^16^, ^17^, ^18^ Monte Carlo (MC) algorithms provide unparalleled physical accuracy and have been applied to many aspects of proton planning and evaluation.^1^, ^2^, ^3^, ^4^, ^5^, ^6^, ^7^, ^8^, ^9^, ^10^, ^11^, ^12^, ^13^, ^14^ Significant effort has been spent on reducing MC execution times through algorithmic modifications, multi‐threaded CPU implementation,^4^ and, most recently, GPU‐based implementation.^3^, ^5^, ^6^, ^14^ Jia et al. developed gPMC, a GPU‐based MC implementation written in OpenCL, which achieved dose calculation times of 8–17 s for 10^7^ particles.^5^, ^6^ Shan et al. developed virtual particle Monte Carlo (VPMC), incorporating some approximations of secondary particle transport to achieve calculation times of 2.8 ± 2.4 s for patient plans.^3^ Lee et al. developed MOQUI, an open‐source GPU‐based MC algorithm providing nearly identical dose values to TOPAS while reducing IMPT plan calculation time from 11 h to 30 s using a single consumer‐level GPU.^14^ GPU‐based MC algorithms have become available in commercial treatment planning systems (TPSs), providing similar or better calculation times using enterprise hardware.^19^ Alternatively, pencil beam (PB) algorithms represent simplified proton dose calculation approaches, which make physical approximations compared to MC but further reduce execution times with standard GPUs.^16^, ^17^, ^18^ Da Silva et al. implemented a GPU‐based double‐Gaussian proton PB algorithm enabling IMPT plan calculation in 0.24 s.^16^

Beyond accurate dose calculation and fast execution times, a key characteristic of proton dose engines for research and development is the ease with which the dose engines can be incorporated into new software applications and the efficiency with which the applications can be created, tested, and modified. This is particularly true for research in proton plan optimization and adaptive planning,^20^ which benefits directly from fast calculation times and the ability to efficiently develop and test algorithms. Python is a high‐level, interpreted programming language known for its simplicity and readability. It has an extensive standard library and open‐source community providing state‐of‐the‐art functionality for data analysis, image processing, and deep learning, among many other domains. These libraries are typically organized in “packages,” which are easily installed and included in new projects through simple import statements, many of which are available through the Python Package Index (PyPI), which currently contains >0.5 million projects. This package repository has been a driving force behind much of the new software developed in recent years by providing easy access to a vast array of packages, facilitating collaboration, and enabling developers to build on each other's work. A Python package that enables fast proton dose calculation would be highly valuable for research and development in IMPT planning. It would allow the extensive advancements made in open‐source software to be effectively applied to clinical IMPT planning challenges. In contrast, existing GPU‐based MC or PB proton dose calculation algorithms have required the use of parallel computing platforms such as CUDA (Nvidia, Santa Clara, CA) or OpenCL, which are compiled languages requiring additional configuration to be incorporated in custom applications, creating a high barrier‐to‐entry and slowing the integration of the latest open‐source libraries.

To this end, the objective of this study was to develop a GPU‐based PB algorithm, which sacrifices some physical accuracy compared to MC for IMPT planning research applications requiring a simple, widely compatible, and sub‐second proton dose calculation solution. We have developed a Python package for proton dose calculation that provides the functionality of a fast GPU‐based PB algorithm, which was accomplished by writing and compiling CUDA code using a CPython wrapper. This paper describes the components of the PB algorithm, including beam modeling for our synchrotron‐based proton delivery system and validation of the package through comparison with measurements and MC calculation for pristine Bragg peaks, spread‐out Bragg peaks (SOBPs), and IMPT plans in patients for various treatment sites.

## 2 METHODS

### 2.1 PB algorithm

We implemented a GPU‐based double Gaussian‐based PB algorithm following similar parameterization as Da Silva et al., focusing on automated beam modeling, simple interface, and fast execution time.^16^, ^17^ The dose D to a point $\overset{⇀}{x}$ for a single mono‐energetic PB (i.e., one “spot”) was computed as

(1) $$D\;\left(\overset{⇀}{x}\right)=MU\cdot IDD\left(E,z_{w}\right)\cdot K_{t}\left(E,\overset{⇀}{x},\;z_{w}\right)$$

where MU is the monitor units, integrated depth‐dose (IDD) is the integrated depth‐dose curve, E is beam energy, $z_{w}$ is water‐equivalent path length to point $\overset{⇀}{x}$, and $K_{t}$ is the total double Gaussian kernel describing the lateral extent of a PB, including central and nuclear halo components. The IDD was saved as a lookup table for each energy as a function of $z_{w}$ with units of cGy mm^2^ / MU. $z_{w}$ was computed using ray tracing as

(2) $$z_{w}\;\left(\overset{⇀}{x}\right)=\int_{z_{0}}^{z}S_{r}\left(z^{\prime }\right)dz\;$$

where $S_{r}$ is the relative linear stopping power to water at distance *z* along the ray from the effective proton source, and $z_{0}$ is the patient surface. The kernel $K_{t}$ was a double Gaussian represented as:

(3) $$\begin{array}{cc} K_{t}\;\left(E,\overset{⇀}{x},\;z\right) & =\left[1-u_{n}\left(R80,z_{w}\right)\right]\;\cdot K_{c}\left(E,\overset{⇀}{x},\;z\right)\\ & \;+u_{n}\left(R80,z_{w}\right)\cdot K_{n}\left(E,\overset{⇀}{x},\;z\right) \end{array}$$

where $u_{n}$ is the nuclear halo fraction, $K_{c}$ is a normalized Gaussian fit to the central portion of the PB, and $K_{n}$ is a normalized Gaussian model of the nuclear halo dose component. The nuclear halo fraction $u_{n}$ was computed using the model described by Soukup et al., which is a function of $z_{w}$ and the proton range given by the R80.^15^ The central Gaussian $K_{c}$ was computed as

(4) $$K_{c}\;\left(E,\overset{⇀}{x},\;z\right)=\frac{1}{2\pi \sigma_{c}^{2}}\;e^{-\;\frac{{\left|\overset{⇀}{x}\right|}^{2}}{2\cdot \sigma_{c}^{2}}}$$

where $|\overset{⇀}{x}|$ is the distance from point x to the PB's central axis, and $\sigma_{c}$ is the sigma providing the central spot width. The central sigma was computed as the sum of two components

(5) $$\sigma_{c}\;\left(z,z_{w}\right)=\sigma_{air}\;\left(z\right)+\sigma_{mcs}\left(z_{w}\right)$$

where $\sigma_{air}\left(z\right)$ is the spot divergence in air and was parameterized as a quadratic function of distance from the effective source z. The parameters defining $\sigma_{air}\left(z\right)$ were determined for each proton beam energy through a least squared error fit to pristine Bragg peaks exported at varying distances from the source, described further in the beam modeling section. The spot divergence in medium $\sigma_{mcs}\left(z_{w}\right)$ was also saved as a lookup table for each beam energy as a function of $z_{w}$, similar to the handling of IDD. The nuclear halo kernel component $K_{n}$ was computed as

(6) $$K_{n}\;\left(E,\overset{⇀}{x},\;z\right)=\frac{1}{2\pi \sigma_{n}^{2}}\;e^{-\;\frac{{\left|\overset{⇀}{x}\right|}^{2}}{2\cdot \sigma_{n}^{2}}}$$

Where $\sigma_{n}\left(R80,z_{w}\right)$ is the sigma describing the width of the nuclear halo and was also computed using the model described by Soukup et al. as a function of the beam R80 and $z_{w}$.^15^ Equations ((1), (2), (3), (4), (5), (6)) describe the calculation of a single pristine Bragg peak in the medium using our PB algorithm implementation. This process can be repeated to compute an IMPT plan consisting of multiple individual PBs, then summing the individual beam doses to produce the final dose distribution.

### 2.2 Beam modeling

Each nominal beam energy was modeled using the following 3 components: (1) a lookup table of IDD versus $z_{w}$, (2) a lookup table of $\sigma_{mcs}$ versus $z_{w}$, and (3) the quadratic function fitting parameters for $\sigma_{air}$ as a function of distance from the effective proton source (z). These parameters were determined based on pristine Bragg peaks computed in a digital water phantom using the MC algorithm in our clinical TPS, RayStation 2023B (RaySearch Labs, Stockholm, Sweden), modeled to match our synchrotron‐based Probeat PBS delivery system (Hitachi, Tokyo, Japan) with 98 discrete energies from 70.2 to 228.7 MeV, similar to the system commissioned by Azcona et al.^21^ This proton delivery system incorporates a nozzle‐mounted range shifter to treat targets extending more shallow than 3.9 cm water equivalent thickness (WET); however, we did not yet model the range shifter in our PB algorithm, discussed further in the Methods and Discussion.

The fitting parameters for $\sigma_{air}$ were determined for each individual energy by computing pristine Bragg peaks at multiple distances from the effective proton source. The effective proton source was defined as the point mid‐way between the x and y scanning magnets. Distances were chosen to place gantry isocenter on the phantom surface (i.e., SSD setup), and positions closer to the source by 10, 20, and 25 cm. For our proton nozzle, this corresponded to four effective SSDs of 163.5, 153.5, 143.5, and 138.5 cm. We focused on SSDs ≤163.5 cm based on the typical clinical practice of placing isocenter within the patient/target rather than treating with extended SSDs. These pristine Bragg peaks were computed for each energy with 1 mm isotropic dose grid, 0.5% statistical uncertainty, and constant 0.2 MU per spot. Following dose calculation, the physical dose grids were exported from the TPS to derive the modeling parameters for each energy. A Python script was written to automate this dose calculation and dose grid export for all 98 energies in our TPS.

Following export, the dose grids were analyzed to compute IDD versus $z_{w}$ at 1 mm resolution and normalized by the delivered MUs to create IDD lookup tables with units of cGy mm^2^ / MU. With this convention, the IDD inherently included the reference dose calibration for each beam energy. The IDD has very little dependence on SSD, but we took the final IDD as the average across all exported SSDs. The R80 for each energy was derived from each final IDD.

The lateral spot profile components $\sigma_{air}$ and $\sigma_{mcs}$ were then determined by fitting a single 2D Gaussian distribution to pristine Bragg peak cross sections at depth increments of 1 mm, providing tables of $\sigma_{c}$ versus $z_{w}$ for each SSD. A reference depth $z_{w,ref}$ was defined, and $\sigma_{c}\left(z_{w,ref}\right)$ was computed for each SSD. $\sigma_{air}\left(z\right)$ was then determined by fitting a quadratic function to $\sigma_{c}\left(z_{w,ref}\right)$ versus distance to the effective proton source (z) across the four SSDs. The lookup table defining $\sigma_{mcs}\left(z_{w}\right)$ was determined by taking the average of $\sigma_{c}\left(z_{w}\right)-\sigma_{c}\left(z_{w,ref}\right)$ across the four SSDs. In this study, we defined $z_{w,ref}$ to be 0.7R80, which was found to be a stable fitting point and improved the lateral spot profile accuracy at depths near the Bragg peak across beam energies. All fitting parameters and lookup table data were automatically derived from the physical dose grids and saved as .csv files labeled by energy index. A master list of energies was saved as a separate .csv containing mappings from energy labels in the plan DICOMs to the lookup table indices.

### 2.3 GPU implementation

**FIGURE 1:**
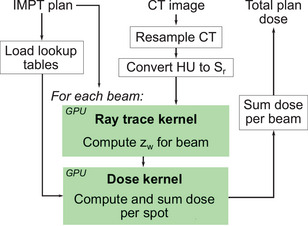
Flow chart of major steps in the pencil beam (PB) algorithm. Black‐outlined steps were executed on the CPU, and green‐highlighted steps were executed on the GPU.

A flowchart of the GPU implementation of the PB algorithm is shown in Figure 1. Since raytracing is only required once per beam angle, this step was implemented as a separate CUDA kernel, taking a NumPy array of the $S_{r}$ value of each voxel as input and returning a corresponding NumPy array containing $z_{w}$ values of each voxel for the given beam and couch angle. HU to $S_{r}$ conversion was performed using Python code based on an HU to $S_{r}$ table produced for our CT scanner using the stoichiometric calibration method.^22^, ^23^ The CUDA kernel only currently supports isotropic voxel dimensions, so the Python pre‐processing code enables CT re‐sampling with user‐specified isotropic voxel dimensions. This voxel dimension is then retained for raytracing and dose calculation, so CT resampling effectively defines the dose grid dimensions in the remainder of the algorithm. After copying the $S_{r}$ array to global GPU memory, we assigned one thread per voxel and conducted raytracing from each voxel location towards the effective proton source (i.e., back‐projection). Raytracing terminates for each voxel when the edge of the image is reached. This approach leads to some redundant calculations between threads for voxels laying on the same ray, but this was not observed to cause performance issues given the parallel implementation. To mitigate sampling artifacts in $z_{w}$ and approximate tortuous paths of protons through tissue, we performed smoothing of $z_{w}$ perpendicular to the beam direction using a uniform distribution with 5 mm radius for voxels with $z_{w}$ values >5 mm, or with radius equal to $z_{w}$ for voxels with $z_{w}$ values ≤5 mm.

A second CUDA kernel was defined to compute the dose associated with a list of spots for a given beam angle. The inputs for the PB kernel were the beam angle, couch angle, pre‐computed $z_{w}$ array, and lists of spot positions (x and y), MUs, and energies. The kernel then returned a NumPy array containing the total physical dose for the beam. The kernel was initialized by copying the $z_{w}$ array and lookup table data to global GPU memory. Threads were initialized to assign N threads per voxel, where N was equal to the number of energy layers in the beam. Within each thread, z was computed and $z_{w}$ was read from memory and used to interpolate the IDD and $\sigma_{mcs}$ lookup tables and compute the values of $\sigma_{air}$, the nuclear halo fraction $u_{n}$, and nuclear halo sigma $\sigma_{n}$ for the corresponding voxel and energy. Each thread then looped through each spot in the list with corresponding energy, computing the distance to the central spot axis $|\overset{⇀}{x}|$ enabling calculation of all terms in Equation 1, which were summed and saved to global memory with atomic addition. The dose was then copied from global GPU memory to host memory and returned as a NumPy array. For plans consisting of multiple beams, the individual beam doses were summed outside of the kernel using NumPy.

### 2.4 Dose engine validation

The PB algorithm was implemented on a Dual workstation (Lambda Labs, San Francisco, CA) with a 10‐core i9 processor (Intel, Santa Clara, CA), 128 GB of RAM, and two Titan RTX GPU (Nvidia, Santa Clara, CA) with 24 GB of VRAM each; however, the PB algorithm only used one GPU per execution. A portion of the validation involved comparing computation times of the PB algorithm to the GPU‐based MC algorithm in our clinical TPS, which was implemented on our clinical servers with RTX 8000 GPUs (Nvidia, Santa Clara, CA) with 48 GB of VRAM each. Although the GPU models and VRAM differed between the MC and PB approaches, both GPUs provide the same 4608 CUDA cores and 130 TFLOPs of single precision performance.

#### 2.4.1 Pristine Bragg peaks

Pristine Bragg peaks were computed for each energy in a digital water phantom using the PB and MC approaches with 1 mm isotropic dose grid voxels. The dose was computed with the isocenter on the phantom surface (SSD = 167.5 cm), 20 cm upstream towards the source (SSD = 147.5 cm), and 20 cm downstream from the source (SSD = 187.5 cm). Lateral spot profiles were extracted from the water surface for each energy, and the profiles were characterized by the standard deviation (SD) of the single Gaussian distribution fit to the lateral spot profile, referred to as “sigma,” which represents a measurement of the lateral spot width. Sigma was determined at the isocenter (iso), upstream of the isocenter by 20 cm (upstream 20 cm), and downstream of the isocenter by 20 cm (downstream 20 cm). These values were compared to sigma values measured using an XRV‐2000 scintillation detector (Logos Systems, Scotts Valley, CA) positioned at the same SSDs. To account for the anisotropy of the measured spot, we computed the mean sigma between the *x*‐and *y*‐directions to compare to the MC and PB algorithms. IDDs were also computed for each pristine Bragg peak using the PB and MC approaches. These calculated IDDs were compared to IDDs measured using a Type 34070 Bragg Peak Chamber (PTW, Freiburg Germany) in a scanning water tank with 0.2 mm resolution. The comparison was performed in terms of the depth in water at which the dose is 80% of the maximum dose beyond the Bragg peak, referred to as the “R80.” All analyses of pristine Bragg peaks were performed in terms of physical dose, and all pristine Bragg peaks computed using the MC algorithm were performed using 0.5% statistical uncertainty.

#### 2.4.2 SOBPs

A reference SOBP was computed using the PB and MC approaches in a digital water phantom, which provides a homogeneous physical dose of 2 Gy in the plateau region when delivered with 200 MU. The plateau region of the SOBP had dimensions of 10 × 10 × 10 cm^3^ and a distal range of 20 cm WET. The SOBP was computed using a 2 mm isotropic dose grid and 0.5% statistical uncertainty using the MC algorithm. The absolute physical depth‐dose curve was extracted from each dose distribution and compared to a measured depth‐dose curve acquired using an Advanced Markus Chamber (PTW, Freiburg Germany) and scanning water tank. The chamber was cross‐calibrated to a farmer chamber, and temperature and pressure corrections were applied to compare absolute depth‐dose values. The percent differences between calculation and measurement were computed for the PB and MC approaches in the plateau region of the SOBP. To assess the performance of the PB algorithm in the presence of heterogeneities, the SOBP was recomputed in a digital water phantom with 5 cm thick lung (HU = ‐700) and bone (HU = 500) inserts with tissue interface parallel with the beam entry direction, mimicking the phantom used by Jia et al. for MC algorithm validation.^6^ The reference SOBP was recomputed in this heterogeneous phantom, and lateral dose profiles were compared at depths within and distal to the heterogeneities.

#### 2.4.3 Patient plans

The PB algorithm was used to recompute IMPT plans optimized in our clinical TPS for four previously treated patients, including prostate, esophageal, brain, and lung cancer patients. Our Institutional Review Board (IRB) approved a secondary analysis of the retrospective anonymized cases. The prostate plan used two lateral beams and a prescription dose of 6000 cGyRBE in 20 fractions. The esophageal cancer case used three posterior beams and a prescription of 5040 cGyRBE in 28 fractions. The brain cancer case used two lateral beams and a prescription of 5040 cGyRBE in 28 fractions. The lung cancer case used two beams (posterior and left lateral) and a prescription of 6600 cGyRBE in 33 fractions. All plan optimization and final dose calculations were performed using the MC algorithm in our TPS. All plans were optimized robustly using ± 3.5% range uncertainty, and the final MC dose was computed using 0.5% statistical uncertainty. The prostate and esophagus cases used 5 mm isotropic setup uncertainty and 3 mm dose grid, and the brain plan used 3 mm isotropic setup uncertainty and 2 mm dose grid. The lung plan employed active breath hold for motion management and used 5 mm setup uncertainty in the superior/inferior direction, 3 mm setup uncertainty in the right/left and anterior/posterior direction, and 3 mm dose grid. Additional beam calculation details are provided in Table 1. While our clinical practice involves the use of a nozzle‐mounted range shifter to treat targets extending to depths <3.9 cm WET, we did not model the range shifter in the PB algorithm in this study, so nozzle‐mounted range shifters were not used for any of the beams in the patient cases selected for validation.

**TABLE 1:** Patient plan beam calculation details.

Plan	Beam number	Gantry angle	Number of energy layers	Number of spots	Central axis air gap (cm)	Relative lateral spot spacing	Monitor units/fraction
Prostate	1	90	29	6768	22.5	0.6	61.45
	2	270	26	6675	21.8	0.6	61.21
Esophagus	1	150	71	14 889	29.2	0.8	95.45
	2	180	68	14 119	30.5	0.8	92.32
	3	210	72	13 508	29.1	0.8	92.41
Brain	1	270	42	4791	30.0	0.6	30.72
	2	90	43	4871	29.8	0.6	30.46
Lung	1	175	38	4550	24.7	0.7	37.42
	2	85	43	4140	27.9	0.7	30.88

Following optimization and final MC dose calculation in the TPS, the plans were recomputed using the PB algorithm and compared to the clinical MC dose in terms of 3D gamma passing rates with 3%/3 mm and 2%/2 mm criteria, using absolute relative biological effectiveness (RBE) dose, the global dose maximum as reference, and considering points with values >10% of the global dose maximum (i.e., threshold value of 10%). The PB dose was also saved as DICOM files and imported into our TPS, and clinical dose volume histogram (DVH) metrics were compared with the MC dose, including mean doses (D_mean_), doses to critical volumes (D_xx_), and volumes receiving critical doses (V_xx_). Dose differences were computed as a percent relative to the prescription. The comparison included CTV D_99%_ and five high‐priority clinical normal tissue metrics for each case. All analysis of patient plans was performed in terms of effective dose following application of a uniform RBE of 1.1.

For the lung cancer case, the beams were also compared to measurements using a patient IMPT quality assurance workflow. Each beam was recomputed in a digital water phantom with gantry angle set to 0°, allowing comparison of planar dose distributions for the MC and PB approaches to measurements acquired using an Octavius ion chamber array (PTW, Freiburg Germany) at a matched depth within the high dose region, selected as 6 cm WET for both beams. Calculated and measured doses were compared using Gamma analysis with 3%/3 mm pass rates, absolute physical dose, global dose maximum as reference, and considering points with dose >10% of the global maximum (i.e., the threshold value was set to 10%). Gamma analysis was performed using VeriSoft 7.2 (PTW, Freiburg Germany).

## 3 RESULTS

Following the compilation of the PB algorithm as a Python package, the code was included in new projects through a simple import statement, with instructions for installation provided in Appendix A.

### 3.1 Pristine Bragg peaks

**FIGURE 2:**
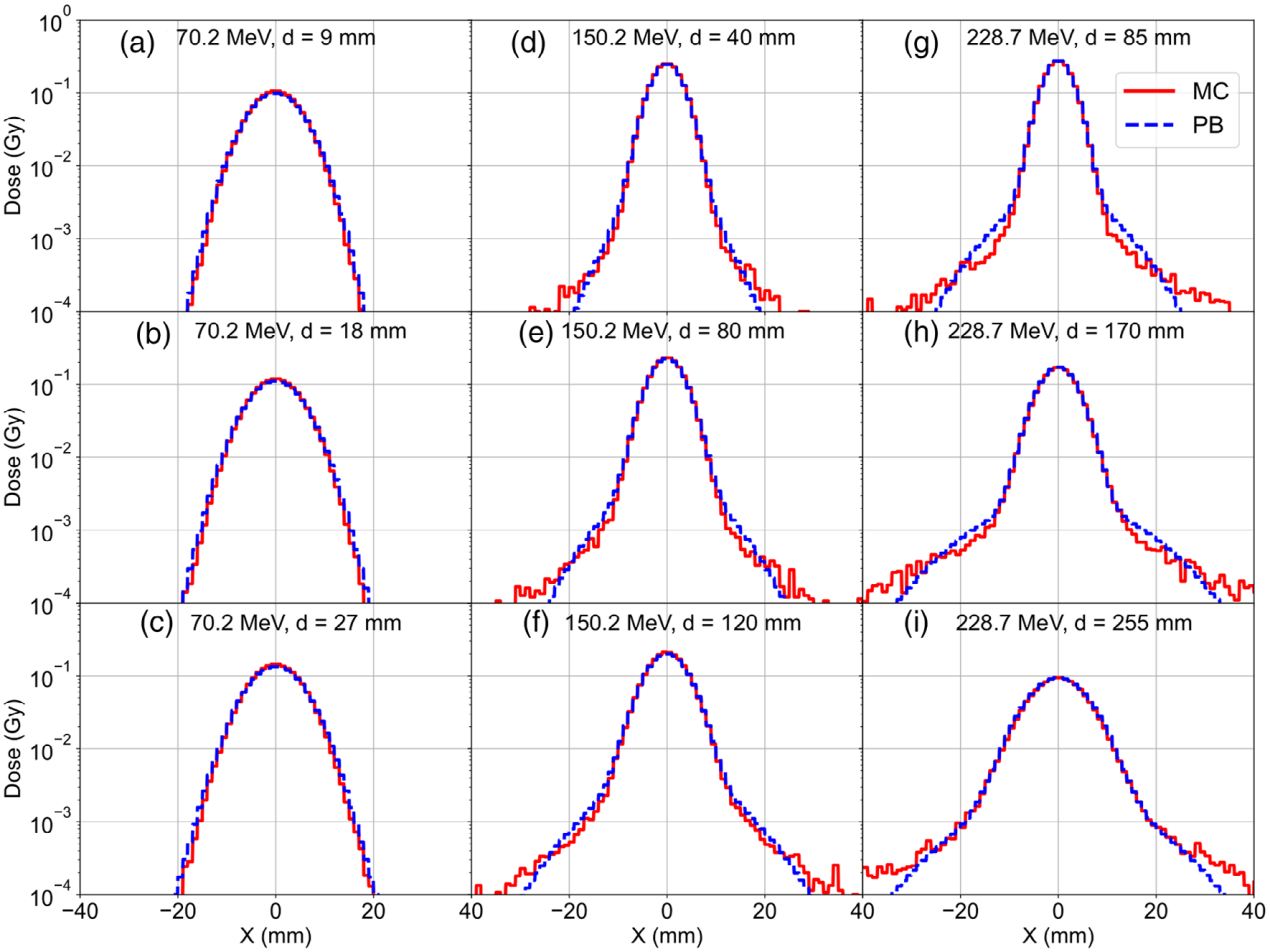
Semilog plots of lateral dose profiles for pristine Bragg peaks in water at varying depths (d) for 70.2 (a–c), 150.2 (d–f), and 228.7 MeV (g–i) comparing the central dose and nuclear halo of the MC and PB approaches for a single spot with 0.2 MU. MC, Monte Carlo; PB, pencil beam.

**FIGURE 3:**
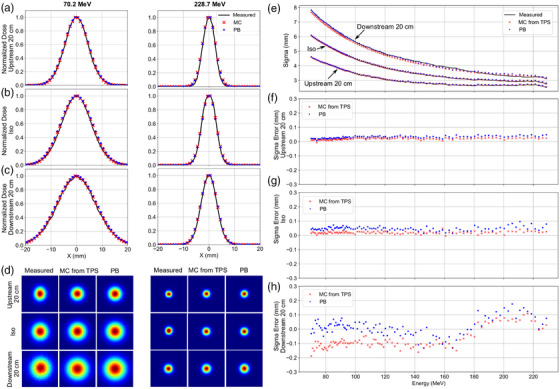
(a–c) Lateral spot profiles in air for 70.2 and 228.7 MeV beam energies at locations upstream of isocenter by 20 cm, at isocenter (iso) and downstream of isocenter by 20 cm computed using the MC and PB methods and compared to measurements acquired using a scintillation detector. (d) Normalized intensity distributions of the spots at each location comparing the measurement with MC and PB calculation approaches. (e) Scatter plot of sigma versus beam energy at each location for the MC, PB, and measurements. (f‐h) Scatter plots the sigma error for the MC and PB approaches compared to the measurement at locations upstream of isocenter by 20 cm, at isocenter (iso), and downstream of isocenter by 20 cm. MC, Monte Carlo; PB, pencil beam.

Example lateral spot profiles are shown in Figure 2, computed using the PB and MC algorithms for three energies at varying depths in a digital water phantom. The semilog plots show the central and nuclear halo components for higher energies and greater depths, matching the trends in the nuclear halo computed using MC. Example lateral profiles at the phantom surface are shown in Figure 3a–c, computed using the PB and MC algorithms and measured using a scintillation detector for the lowest and highest energies for the location at isocenter (iso), upstream of isocenter by 20 cm (Upstream 20 cm) and downstream of isocenter by 20 cm (Downstream 20 cm).

Normalized intensity images of the pristine Bragg peak cross sections are shown in Figure 3d. The profiles and intensity distributions illustrate the spot divergence in air. Fitted sigma values for the MC, PB, and measurements are shown in Figure 3e. The sigma errors, computed as the difference between modeled and measured sigma values, for the MC and PB approaches are shown in Figure 3f–h for the locations upstream of isocenter by 20 cm, at isocenter, and downstream of isocenter by 20 cm, respectively. The mean ± SD across energies for the MC approach were 0.02 ± 0.01, 0.03 ± 0.01, and −0.07 ± 0.07 mm for the locations upstream of isocenter by 20 cm, at isocenter, and downstream of isocenter by 20 cm, respectively. The mean ± SD for the PB algorithm were 0.03 ± 0.01, 0.05 ± 0.01, and 0.01 ± 0.06 mm for the locations upstream of isocenter by 20 cm, at isocenter, and downstream of isocenter by 20 cm, respectively.

**FIGURE 4:**
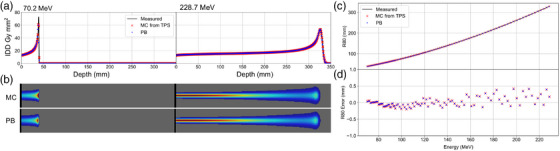
(a) IDD curves for 70.2 and 228.7 MeV beam energies in water computed using MC and PB approaches and compared to measurement using a water tank and Bragg Peak chamber. (b) Pristine Bragg peak dose distributions were computed using the MC and PB approaches for 70.2 and 228.7 MeV beam energies. (c) Scatter plot of R80 versus beam energy for the MC and PB approaches compared to measured R80s using a Bragg peak chamber. (d) R80 errors of the MC and PB approaches compared to measurements. IDD, integrated depth‐dose; MC, Monte Carlo; PB, pencil beam.

Example IDDs comparing MC, PB, and measurement using a water tank and Bragg peak chamber are shown in Figure 4a for 70.2 and 228.7 MeV beam energies. Figure 4b shows dose distributions for a single pristine Bragg peak in a digital water phantom produced using MC and PB approaches. Figure 4c shows a scatter plot of R80s versus energy for MC, PB, and measurements, and Figure 4d shows a scatter plot of R80 error of the MC and PB approaches compared to measurements. The MC and PB algorithms showed nearly identical IDDs, and when compared to Bragg Peak Chamber measurements, mean ± SD of R80 differences across all energies for the MC and PB algorithms were 0.0 ± 0.1 and 0.0 ± 0.1 mm, respectively. All R80 errors were within ± 0.5 mm, which was less than the voxel dimensions of the dose grids used for beam modeling and evaluation.

### 3.2 SOBP

**FIGURE 5:**
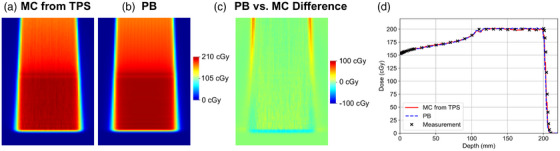
(a–b) Dose distributions computed using the MC and PB approaches in a homogeneous digital water phantom. (c) Dose difference between the PB and MC approaches. (d) Depth dose curves from the central axis of the SOBP were computed using the MC and PB approaches and compared to measurements acquired with an Advanced Markus Chamber and scanner water tank. MC, Monte Carlo; PB, pencil beam; SOBP, spread‐out Bragg peaks.

Dose distributions for the reference SOBP computed using the MC and PB approaches in a homogeneous digital water phantom are shown in Figures 5a,b. The difference between the PB and MC dose distributions is shown in Figure 5c. The PB algorithm agreed well with the MC algorithm in the center of the SOBP but slightly overestimated the lateral penumbra in the entrance region and slightly underestimated the proton range. Figure 5d compares the depth‐dose profiles computed using the MC and PB approaches with the Advanced Markus Chamber measurements. In the plateau region, differences between calculation and measurement were −0.1 ± 1.0% and 0.4 ± 1.1% for the MC and PB approaches, respectively.

**FIGURE 6:**
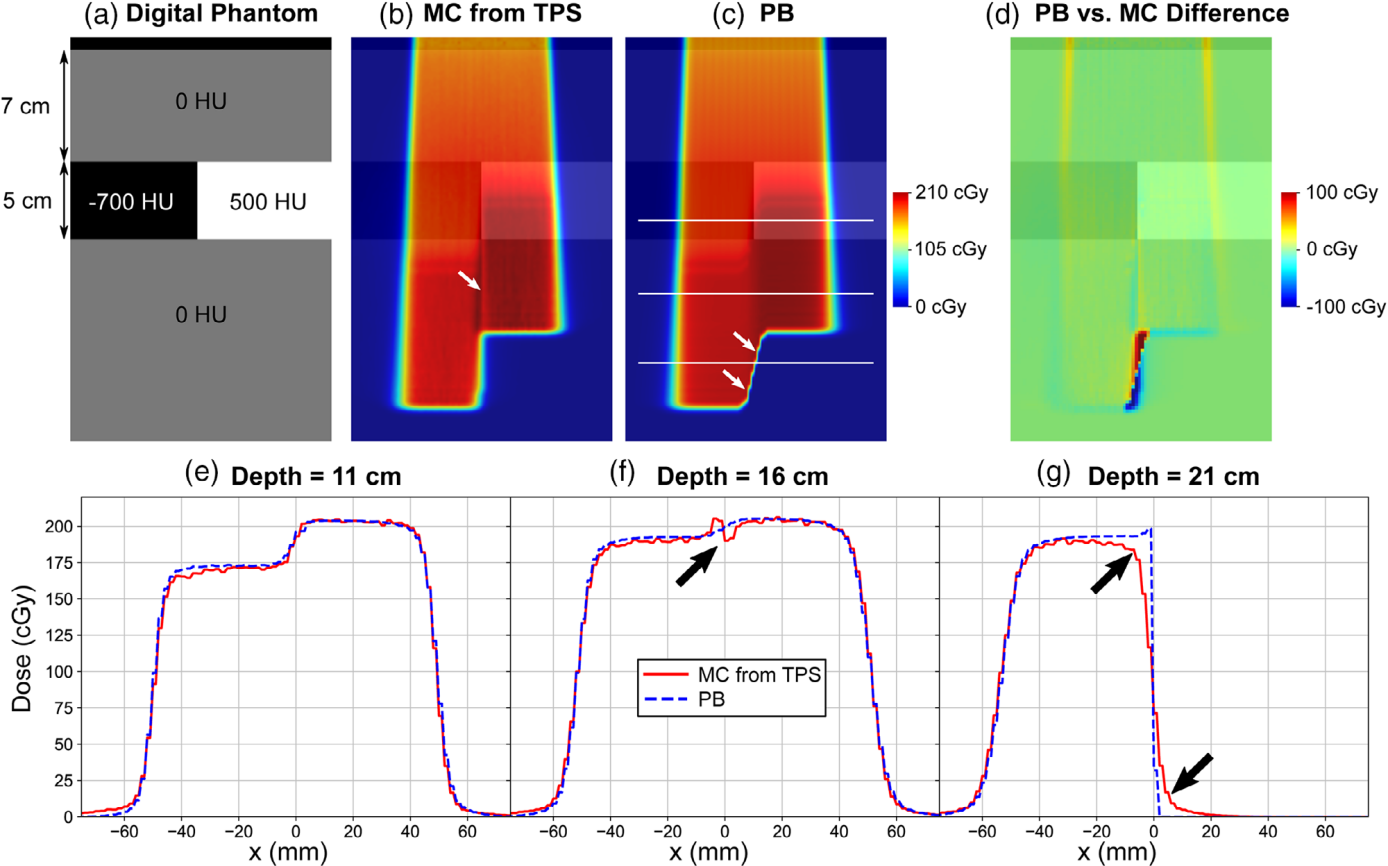
(a) Digital phantom incorporating 5 cm thick lung and bone heterogeneities at 7 cm depth. (b–c) Reference SOBP computed using the MC and PB approaches in the heterogeneous phantom. Horizontal lines in (c) indicate the depths of the compared lateral profiles. (d) Dose difference between the PB and MC approaches. (e–g) Lateral dose profiles comparing MC and PB approaches. Arrows highlight areas of discrepancy between the PB and MC approaches. MC, Monte Carlo; PB, pencil beam; SOBP, spread‐out Bragg peaks

The heterogeneous digital phantom is shown in Figure 6a. Dose distributions for the SOBP computed in the heterogeneous phantom using the MC and PB approaches are shown in Figure 6b,c, respectively. The dose difference between PB and MC is shown in Figure 6d, and lateral dose profiles at depths of 11, 16, and 21 cm are shown in Figure 6e–g. Proximal and inside of the heterogenous region, the dose distributions agreed well. However, beyond the heterogeneous region, discrepancies are observed in the region immediately beyond the boundary between the lung and bone inserts. First, the PB algorithm does not model the small hot and cold spots caused by lateral proton scatter beyond the tissue boundary captured by the MC algorithm, highlighted in Figure 6f. Second, the PB algorithm showed an unrealistic abrupt lateral penumbra beyond the heterogeneity shown in Figure 6g. This is attributed to the inability of the PB algorithm to model lateral proton scatter beyond large heterogeneity, particularly near the Bragg peak.

### 3.3 Patient plans

**FIGURE 7:**
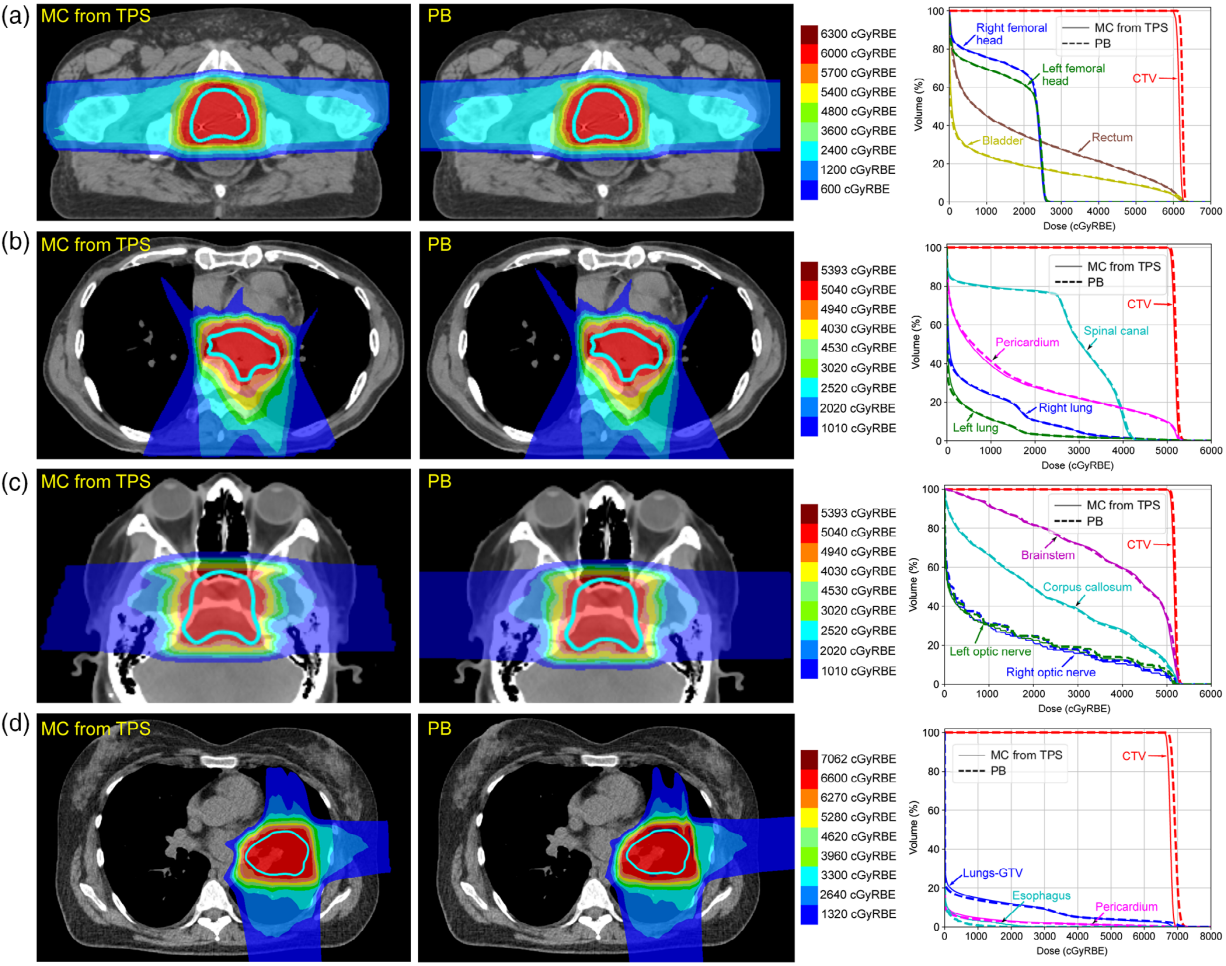
Example isodose distributions computed using the MC algorithm from the TPS, PB algorithm, and associated DVH curves for (a) a prostate cancer patient, (b) an esophageal cancer patient, (c) a brain cancer patient, and (d) a lung cancer patient. DVH, dose volume histogram; MC, Monte Carlo; PB, pencil beam; TPS, treatment planning system.

Computation times of the PB algorithm were 0.23, 0.39, 0.27, and 0.23 s for the prostate, esophagus, brain, and lung cases, respectively, compared to 3.19, 8.68, 3.74, and 3.12 s for the commercial GPU‐based MC algorithm. Isodose distributions and DVH for the four test patient cases are shown in Figure 7. Mean ± SD gamma passing rates at 3%/3 and 2%/2 mm were 98.6 ± 2.3% and 96.0 ± 5.1%, respectively. DVH metrics of interest are provided in Table 2. The PB algorithm tended to slightly overestimate dose metrics compared to the MC algorithm with a mean ± SD percent difference of 0.5 ± 3.6%. In particular, the PB algorithm slightly overestimated CTV D_99%_ for all four cases. For the esophagus and lung cancer cases, the point maximum PB doses were computed at the distal edge of the beams outside of the CTV, attributed to issues in modeling proton scatter near the Bragg peak in heterogeneous regions. The largest errors were observed for the lung cancer case, including underestimation of the maximum esophagus dose (D_0.03cc_) of 13.8%, also attributed to PB algorithm accuracy degradation in the presence of significant heterogeneities. When comparing computed dose to ionization chamber array measurements for the two beams comprising the lung cancer plan, the MC and PB approaches provided mean Gamma passing rates of 100.0% and 99.7%, respectively, at 3%/3 mm criteria. Gamma distributions are provided in the Supplemental Material.

**TABLE 2:** DVH metrics and percent differences between MC and PB approaches.

Plan	Structure	Metric	MC from TPS	PB	% Difference
Prostate (300 cGyRBE x 20 fx)	CTV	D_99%_ (cGyRBE)	6016	6142	2.1
	Body	D_0.03 cc_ (cGyRBE)	6366	6313	−0.9
	Rectum	D_0.03 cc_ (cGyRBE)	6285	6273	−0.2
	Bladder	V_5775 cGyRBE_ (%)	5.3	5.0	−0.3
	L femoral head	D_0.3 cc_ (cGyRBE)	2695	2681	−0.2
	R femoral head	D_0.3 cc_ (cGyRBE)	2639	2646	0.1
Esophagus (180 cGyRBE × 28 fx)	CTV	D_99%_ (cGyRBE)	5053	5089	0.7
	Body	D_0.03 cc_ (cGyRBE)	5354	5572	4.3
	Spinal canal	D_0.03 cc_ (cGyRBE)	4340	4371	0.6
	Pericardium	D_mean_ (cGyRBE)	1456	1483	0.5
	L lung	V_2000 cGyRBE_ (%)	10.6	10.6	0.0
	R lung	V_2000 cGyRBE_ (%)	3.4	3.4	0.0
Brain (180 cGyRBE × 28 fx)	CTV	D_99%_ (cGyRBE)	5049	5083	0.7
	Body	D_0.03 cc_ (cGyRBE)	5319	5349	0.6
	Brainstem	D_0.03 cc_ (cGyRBE)	5302	5315	0.3
	Corpus callosum	D_mean_ (cGyRBE)	2312	2298	−0.3
	L optic nerve	D_0.03 cc_ (cGyRBE)	4854	4978	2.5
	R optic nerve	D_0.03 cc_ (cGyRBE)	5060	5131	1.4
Lung (200 cGyRBE × 33 fx)	CTV	D_99%_ (cGyRBE)	6637	6733	1.5
	Body	D_0.03 cc_ (cGyRBE)	6992	7464	7.2
	Esophagus	D_0.03 cc_ (cGyRBE)	2594	1684	−13.8
	Lungs−GTV	V_2000 cGyRBE_ (%)	12.0	11.5	−0.5
	Lungs−GTV	D_mean_ (cGyRBE)	586	571	−0.2
	Pericardium	D_0.03 cc_ (cGyRBE)	6912	7257	5.2

Abbreviations: DVH, dose volume histogram; MC, Monte Carlo; PB, pencil beam; RBE, relative biological effectiveness; TPS, treatment planning system.

## 4 DISCUSSION

In this study, we developed a Python package for GPU‐based proton dose calculation, which implements a double Gaussian‐based PB algorithm enabling plan calculation in 0.28 ± 0.07 s through a simple Python interface compatible with an extensive and growing ecosystem of open‐source libraries. While many open‐source proton dose calculation algorithms exist, particularly MC dose engines, these algorithms have typically been implemented as standalone applications using compiled code such as CUDA and C. These tools provide sophisticated physical modeling and high computational performance, particularly in recent multi‐threaded CPU and GPU implementations; however, integrating these tools into new applications is challenging, and execution times remain higher than those available from GPU‐accelerated PB algorithms. While these characteristics suit many proton research applications, slow execution times and complex integration with custom software applications present specific challenges for IMPT plan optimization research. A single IMPT plan may consist of >10^4^ individual spots, and robust optimization requires repeated dose calculation under uncertainty scenarios, making proton dose calculation time crucial to the clinical use of these algorithms, which currently can take >1 h to execute for complex cases with large targets.^24^ Moreover, there is growing interest in advanced and automated inverse IMPT optimization approaches, building upon advances in machine learning and deep learning^25^ which are developed and tested most efficiently using open‐source libraries such as PyTorch or Tensorflow. Fast and high‐quality automated IMPT planning could lead to significant clinical advances, such as facilitating rapid or online adaptive treatment planning and overcoming issues with the sensitivity of IMPT plans to patient tissue changes.

Our PB algorithm achieved 0.28 ± 0.07 s execution time for patient IMPT plans for a variety of treatment sites. This is faster than the MC algorithm in our clinical TPS and recent fast GPU‐based MC implementations. The hardware used to implement the MC and PB algorithms in this study was not identical, which is a limitation of the comparison of computation times, but the GPU specifications were similar, and the computation times matched trends in the literature. Da Silva et al. reported execution times of 0.24 s for clinical head and neck cases using a CUDA implementation of a double Gaussian‐based PB algorithm,^16^ similar to our performance. Beyond PB algorithms, recent investigations into accelerated proton dose calculation using deep learning have been proposed to enable further reductions in execution times and improvements in accuracy by training a deep learning network to predict proton PB dose given an input region of tissue densities.^26^, ^27^ However, these approaches still require 10–15 s to compute patient IMPT plans, and modeling remains complex, requiring network re‐training for each beam model, making widespread adoption challenging.^27^

Our PB algorithm has several limitations in terms of physical accuracy. Similar to other PB implementations, our algorithm does not simulate individual particle interactions, so it suffers from limited accuracy in the presence of significant tissue heterogeneities such as those observed in the thorax,^28^ where we observed the largest dose metric errors. A comparison of the dose calculated in a heterogeneous digital phantom revealed limitations in the ability of the PB algorithm to account for lateral proton scatter distal to the heterogeneity, which is a limitation of the approach. We also implemented a simple polynomial and lookup table‐based modeling of spot divergence in air and the medium rather than modeling the characteristic radiation scatter angle in air and tissue. This choice again simplified the beam modeling and PB algorithm implementation but likely contributed to the small errors in sigma we observed in our validation work and would contribute to errors in spot width in heterogeneous tissues. Additionally, our PB algorithm did not decompose individual PBs into smaller computational PBs, as has been performed in previous studies.^15^, ^17^ This choice was made based on our PB kernel design, which assigned threads to each dose grid voxel, thereby allowing water equivalent path length to be modeled for each voxel rather than following the central axes of a collection of computational PBs. This choice also simplified the kernel design and reduced execution times while maintaining sufficient physical accuracy in the presence of heterogeneities, which was consistent with the overall design objectives of the algorithm. Previous studies have also demonstrated the benefits of using a triple‐Gaussian PB model to better model the nuclear halo compared to the double‐Gaussian we employed.^29^ We also chose the double‐Gaussian model to maintain model simplicity and minimize computation time, but implementing the triple‐Gaussian model could improve accuracy in future versions. Finally, our clinical practice involves a nozzle‐mounted range shifter to treat targets extending shallower than 3.9 cm WET. However, we have not yet modeled or validated the range shifter for our PB algorithm, so cannot yet apply it to clinical cases requiring a range shifter. Given the PB algorithm's simple model of spot divergence in the vicinity of the isocenter, we plan to model the range shifter using a new set of lookup tables generated using MC to ensure spot divergence is accurately modeled. This update will require validation with additional measurements and will be the topic of future work.

## 5 CONCLUSIONS

We have developed a Python package providing a fast proton PB algorithm that enables sub‐second clinical plan calculation through a simple import statement and function call. This tool demonstrated sufficient accuracy for many research and development applications, particularly those that benefit from fast execution times and compatibility with open‐source libraries. Limitations of the PB algorithm were identified, which were related to the limited modeling of lateral proton scatter distal to significant heterogeneities, which should be carefully considered. MC remains the gold standard for final clinical dose calculation. The PB algorithm has been released to the proton radiotherapy community as an open‐source tool called “DoseCUDA” to facilitate and accelerate IMPT planning research and development, and several directions for future improvements were identified and discussed.

## AUTHOR CONTRIBUTION STATEMENT

Mahasweta Bhattacharya contributed to the acquisition, analysis, and interpretation of data and drafted the work for important intellectual content. Calin Reamy contributed to the acquisition, analysis, and interpretation of data and drafted the work for important intellectual content. Heng Li contributed to the conception and design of the work and revised the work for important intellectual content. Junghoon Lee contributed to the conception and design of the work and revised the work for important intellectual content. William T.Hrinivich contributed to the conception and design of the work, acquisition, analysis, and interpretation of data and drafted the work for important intellectual content. All authors contributed to the final approval of the version to be published and agree to be accountable for all aspects of the work in ensuring that questions related to the accuracy or integrity of any part of the work are appropriately investigated and resolved.

## CONFLICT OF INTEREST STATEMENT

W.T.H. and J.L. conduct research funded by Varian Medical Systems unrelated to this study. M.B. is currently employed by Sanofi S.A. Work was conducted at JHU. The remaining authors have no relevant conflicts of interest to disclose.

## ETHICS STATEMENT

The retrospective analysis of anonymized patient plans was approved by our Institutional Review Board.

## Supporting information



Supporting Information

## Data Availability

The source code is publicly available in the repository described in the Appendix. Other data is available from the authors upon reasonable request.

## APPENDIX A —DOSECUDA INSTALLATION INSTRUCTIONS

The following illustrates the major steps of downloading and compiling the dose calculation algorithm. Please see the repository homepage for the most up‐to‐date installation prerequisites and instructions.

### A.1 Prerequisites

- Python ≥3.6
- CMake ≥3.15
- CUDA Toolkit (Ensure you have a compatible version for your GPU)
- Git (for cloning the repository)

### A.2 Windows

1. (Optional but recommended): Create a virtual environment. Open the command prompt (cmd) and run:

python ‐m venv dosecuda‐env
dosecuda‐env∖Scripts∖activate

2 Clone the DoseCUDA repository:

git clone https://github.com/jhu‐som‐radiation‐oncology/DoseCUDA.git
cd DoseCUDA

3 Install DoseCUDA using pip from a developer command prompt (e.g. x64 Native Tools Command Prompt):

python ‐m pip install .

4 Verify the installation:

python tests∖test_phantom_impt.py

5 (If used) Deactivate the virtual environment by running:

deactivate

### A.3 Linux

1. (Optional but recommended): Create a virtual environment. Open terminal and run:

python3 ‐m venv dosecuda‐env
source dosecuda‐env/bin/activate

2 Clone the DoseCUDA repository:

git clone https://github.com/jhu‐som‐radiation‐oncology/DoseCUDA.git
cd DoseCUDA

3 Install DoseCUDA using pip:

python ‐m pip install .

4 Verify the installation:

python tests/test_phantom_impt.py

5 (If used) Deactivate the virtual environment by running:

deactivate

## References

[1] Verbeek N , Wulff J , Bäumer C , Smyczek S , Timmermann B , Brualla L . Single pencil beam benchmark of a module for Monte Carlo simulation of proton transport in the PENELOPE code. Med Phys. 2021;48(1):456‐476. doi:10.1002/mp.14598 33217026

[2] Kozłowska WS , Böhlen TT , Cuccagna C , et al. FLUKA particle therapy tool for Monte Carlo independent calculation of scanned proton and carbon ion beam therapy. Phys Med Biol. 2019;64(7):075012. doi:10.1088/1361-6560/ab02cb 30695766

[3] Shan J , Feng H , Morales DH , et al. Virtual particle Monte Carlo: a new concept to avoid simulating secondary particles in proton therapy dose calculation. Med Phys. 2022;49(10):6666‐6683. doi:10.1002/mp.15913 35960865 PMC9588716

[4] Souris K , Lee JA , Sterpin E . Fast multipurpose Monte Carlo simulation for proton therapy using multi‐ and many‐core CPU architectures. Med Phys. 2016;43(4):1700‐1712. doi:10.1118/1.4943377 27036568

[5] Qin N , Botas P , Giantsoudi D , et al. Recent developments and comprehensive evaluations of a GPU‐based Monte Carlo package for proton therapy. Phys Med Biol. 2016;61(20):7347‐7362. doi:10.1088/0031-9155/61/20/7347 27694712 PMC5378691

[6] Jia X , Schümann J , Paganetti H , Jiang SB . GPU‐based fast Monte Carlo dose calculation for proton therapy. Phys Med Biol. 2012;57(23):7783‐7797. doi:10.1088/0031-9155/57/23/7783 23128424 PMC4474737

[7] Guan F , Peeler C , Bronk L , et al. Analysis of the track‐ and dose‐averaged LET and LET spectra in proton therapy using the geant 4 Monte Carlo code. Med Phys. 2015;42(11):6234‐6247. doi:10.1118/1.4932217 26520716 PMC4600086

[8] Jarlskog CZ , Paganetti H . Physics settings for using the Geant4 toolkit in proton therapy. IEEE Trans Nucl Sci. 2008;55:1018‐1025. doi:10.1109/TNS.2008.922816

[9] Prusator M , Ahmad S , Chen Y . TOPAS simulation of the mevion S250 compact proton therapy unit. J Appl Clin Med Phys. 2017;18(3):88‐95. doi:10.1002/acm2.12077 PMC5689857 28444840

[10] Chen Z , Liu H , Zhao J , Kaess S . TOPAS Monte Carlo simulation for a scanning proton therapy system in SPHIC. J Radiat Res Appl Sci. 2022;15(1):122‐129. doi:10.1016/j.jrras.2022.01.016

[11] Liu H , Li Z , Slopsema R , Hong L , Pei X , Xu XG . TOPAS Monte Carlo simulation for double scattering proton therapy and dosimetric evaluation. Physica Medica. 2019;62:53‐62. doi:10.1016/j.ejmp.2019.05.001 31153399

[12] Testa M , Schümann J , Lu HM , et al. Experimental validation of the TOPAS Monte Carlo system for passive scattering proton therapy. Med Phys. 2013;40(12):121719. doi:10.1118/1.4828781 24320505 PMC4109425

[13] Perl J , Shin J , Schümann J , Faddegon B , Paganetti H . TOPAS: an innovative proton Monte Carlo platform for research and clinical applications. Med Phys. 2012;39(11):6818‐6837. doi:10.1118/1.4758060 23127075 PMC3493036

[14] Lee H , Shin J , Verburg JM , et al. MOQUI: an open‐source GPU‐based Monte Carlo code for proton dose calculation with efficient data structure. Phys Med Biol. 2022;67(17):174001. doi:10.1088/1361-6560/ac8716 PMC9513828 35926482

[15] Soukup M , Fippel M , Alber M . A pencil beam algorithm for intensity modulated proton therapy derived from Monte Carlo simulations. Phys Med Biol. 2005;50(21):5089‐5104. doi:10.1088/0031-9155/50/21/010 16237243

[16] da Silva J , Ansorge R , Jena R . Fast pencil beam dose calculation for proton therapy using a double‐Gaussian beam model. Front Oncol. 2015;5:281. doi:10.3389/fonc.2015.00281 26734567 PMC4683172

[17] Da Silva J , Ansorge R , Jena R . Sub‐second pencil beam dose calculation on GPU for adaptive proton therapy. Phys Med Biol. 2015;60(12):4777‐4795. doi:10.1088/0031-9155/60/12/4777 26040956

[18] Fujimoto R , Kurihara T , Nagamine Y . GPU‐based fast pencil beam algorithm for proton therapy. Phys Med Biol. 2011;56(5):1319‐1328. doi:10.1088/0031-9155/56/5/006 21297243

[19] Schreuder AN , Bridges DS , Rigsby L , et al. Validation of the RayStation Monte Carlo dose calculation algorithm using realistic animal tissue phantoms. J Appl Clin Med Phys. 2019;20(10):160‐171. doi:10.1002/acm2.12733 PMC6806482 31541536

[20] Paganetti H , Botas P , Sharp GC , Winey B . Adaptive proton therapy. Phys Med Biol. 2021;66(22):22TR01. doi:10.1088/1361-6560/ac344f PMC8628198 34710858

[21] Azcona JD , Aguilar B , Perales Á , et al. Commissioning of a synchrotron‐based proton beam therapy system for use with a Monte Carlo treatment planning system. Radiation Physics and Chemistry. 2023;204:110708. doi:10.1016/j.radphyschem.2022.110708

[22] Goma C , Almeida IP , Verhaegen F . Revisiting the single‐energy CT calibration for proton therapy treatment planning: a critical look at the stoichiometric method. Phys Med Biol. 2018;63(23):235011. doi:10.1088/1361-6560/aaede5 30474618

[23] Schneider U , Pedroni E , Lomax A . The calibration of CT Hounsfield units for radiotherapy treatment planning. Phys Med Biol. 1996;41(1):111‐124. doi:10.1088/0031-9155/41/1/009 8685250

[24] Buti G , Souris K , Barragán Montero AM , Cohilis M , Lee JA , Sterpin E . Accelerated robust optimization algorithm for proton therapy treatment planning. Med Phys. 2020;47(7):2746‐2754. doi:10.1002/mp.14132 32155667

[25] Zhang G , Zhou L , Han Z , Zhao W , Peng H . SWFT‐Net: a deep learning framework for efficient fine‐tuning spot weights towards adaptive proton therapy. Phys Med Biol. 2022;67(24):245010. doi:10.1088/1361-6560/aca517 36541496

[26] Wu C , Nguyen D , Xing Y , et al. Improving proton dose calculation accuracy by using deep learning. Mach Learn Sci Technol. 2021;2(1):015017. doi:10.1088/2632-2153/abb6d5 35965743 PMC9374098

[27] Pastor‐Serrano O , Perkó Z . Millisecond speed deep learning based proton dose calculation with Monte Carlo accuracy. Phys Med Biol. 2022;67(10):105006. doi:10.1088/1361-6560/ac692e 35447605

[28] Taylor PA , Kry SF , Followill DS . Pencil beam algorithms are unsuitable for proton dose calculations in lung. Int J Radiat Oncol Biol Phys. 2017;99(3):750‐756. doi:10.1016/j.ijrobp.2017.06.003 28843371 PMC5729062

[29] Hirayama S , Takayanagi T , Fujii Y , et al. Evaluation of the influence of double and triple Gaussian proton kernel models on accuracy of dose calculations for spot scanning technique. Med Phys. 2016;43(3):1437‐1450. doi:10.1118/1.4942386 26936728

