# Supplementary material for: A Python package for fast GPU‐based proton pencil beam dose calculation
Source: J Appl Clin Med Phys. 2025 Apr 9;26(6):e70093. doi: 10.1002/acm2.70093 (PMC12148800; doi:10.1002/acm2.70093)
Supplement: Supplementary file 1 — Supporting Information [file ACM2-26-e70093-s001.docx]

**Supplemental Materials – Additional Patient Quality Assurance (QA) Measurement Results**

Gamma distributions are shown in Figure S1, comparing lung plan dose distributions computed using the Monte Carlo (MC) algorithm from our treatment planning system (TPS) and GPU-based pencil beam (PB) algorithm to ionization chamber array measurements acquired using an Octavius device (PTW, Freiburg, Germany). Details of the comparison are described in the Methods section 2.4.3. Gamma pass rates at 3%/3 mm criteria for beams 1 and 2 were 100.0% and 100.0% for the MC algorithm and 99.3% and 100.0% for the PB algorithm.


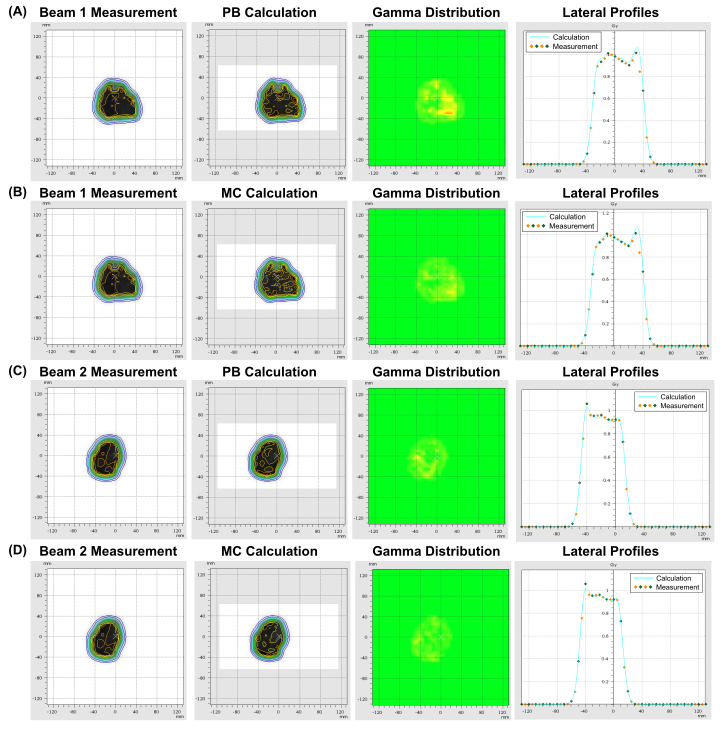


**Figure S1** Screenshots from VeriSoft 7.2 (PTW, Freiburg Germany) showing results of patient IMPT QA using the MC and PB algorithms compared to ionization chamber array measurements for a lung cancer plan consisting of two beams. PB and MC results for beam 1 are shown in (A) and (B), respectively, and PB and MC results for beam 2 are shown in (C) and (D), respectively.
